# Phylogenetic groups and cephalosporin resistance genes of *Escherichia coli *from diseased food-producing animals in Japan

**DOI:** 10.1186/1751-0147-53-52

**Published:** 2011-10-12

**Authors:** Tetsuo Asai, Kaori Masani, Chizuru Sato, Mototaka Hiki, Masaru Usui, Kotaro Baba, Manao Ozawa, Kazuki Harada, Hiroshi Aoki, Takuo Sawada

**Affiliations:** 1 National Veterinary Assay Laboratory, Ministry of Agriculture, Forestry and Fisheries, 1-15-1 Tokura, Kokubunji, Tokyo 185-8511; 2 Nippon Veterinary and Life Science University, 1-7-1 Kyonancho, Musashino, Tokyo 180-8601, Japan

**Keywords:** *Escherichia coli*, food-producing animals, cephalosporin resistance, extended spectrum β-lactamase, AmpC β-lactamase, phylogenetic grouping

## Abstract

A total of 318 *Escherichia coli *isolates obtained from different food-producing animals affected with colibacillosis between 2001 and 2006 were subjected to phylogenetic analysis: 72 bovine isolates, 89 poultry isolates and 157 porcine isolates. Overall, the phylogenetic group A was predominant in isolates from cattle (36/72, 50%) and pigs (101/157, 64.3%) whereas groups A (44/89, 49.4%) and D (40/89, 44.9%) were predominant in isolates from poultry. In addition, group B2 was not found among diseased food-producing animals except for a poultry isolate. Thus, the phylogenetic group distribution of *E. coli *from diseased animals was different by animal species. Among the 318 isolates, cefazolin resistance (minimum inhibitory concentrations: ≥32 μg/ml) was found in six bovine isolates, 29 poultry isolates and three porcine isolates. Of them, 11 isolates (nine from poultry and two from cattle) produced extended spectrum β-lactamase (ESBL). The two bovine isolates produced *bla_CTX-M-2_*, while the nine poultry isolates produced *bla_CTX-M-25 _*(4), *bla_SHV-2 _*(3), *bla_CTX-M-15 _*(1) and *bla_CTX-M-2 _*(1). Thus, our results showed that several types of ESBL were identified and three types of β-lactamase (SHV-2, CTX-M-25 and CTX-M-15) were observed for the first time in *E. coli *from diseased animals in Japan.

## Findings

Pathogenic *Escherichia coli *is the causative agent of colibacillosis that brings severe clinical sighs such as diarrhea, meningitis and sepsis in domestic animals. *E. coli *can be divided into four phylogenetic groups using multiplex PCR method [1]. Several authors have analyzed the distribution of the main phylogenetic groups among *E. coli *strains isolated from human and animals. Extraintestinal pathogenic *E. coli *usually belongs to groups B2 and D, while the commensal strains are groups A and B1 in humans [2]. In healthy food-producing animals, a predominant distribution of group B1 was reported in *E. coli *from cattle, while group A was predominantly prevalent in pigs and chickens [3-5]. On the contrary, although predominant phylogenetic groups were reported in *E. coli *from diseased poultry [6-8], little information regarding to diseased cattle and pigs was available.

Antimicrobial agents have been widely used for treatment of colibacillosis in domestic animals. Antimicrobial resistance is more frequently found in *E. coli *isolates from diseased animals than apparently healthy animals [9]. Increases in cephalosporin-resistant *E. coli *in domestic animals have been a significant worldwide concern including Japan. Extended-spectrum β-lactamase (ESBL)-producing isolates of *E. coli *were identified in domestic animals around 2000 in Japan [10,11]. The third and greater generation cephalosporin antibiotics are clinically important antimicrobial agents in human and animal medicines. Such agents are approved to treat bacterial diseases in cattle and pigs and not licensed for enhancing animal growth in Japan. However, cephalosporin-resistant *E. coli *has been identified in isolates from not only cattle and pigs but also poultry in Japan [10,12]. At present the reason for the prevalence of cephalosporin-resistant *E. coli *in poultry is unclear.

In the present study, we determined the distribution of phylogenetic groups in *E. coli *from food-producing animals affected with colibacillosis in Japan. In addition, we examined the characteristics of cephalosporin-resistant isolates of *E. coli *from the diseased animals.

A total of 318 *E. coli *isolates from different animals affected with colibacillosis between 2001 and 2006 throughout Japan were used in this study: 72 bovine isolates, 89 poultry isolates and 157 porcine isolates in 23, 20 and 28 prefectures, respectively, located from north to south Japan. All the strains were stored in 10% skimmed milk at -80°C until use. The phylogenetic grouping was performed by multiplex PCR as described by Clermont et al. [1].

The minimum inhibitory concentration (MIC) of cefazolin (CEZ) was determined by an agar dilution method in accordance with the guidelines of the Clinical Laboratory Standards Institute (CLSI) [13,14]. The MIC of some of the isolates to CEZ was previously determined [15]. Susceptibilities of CEZ-resistant isolates to several β-lactamase antibiotics, cefpodoxime, cefotaxime, ceftazidime, aztreonam, imipenem and meropenem were tested according to the CLSI guideline [13,14] using the commercially available broth microdilution test (Eiken Co. LTD, Tokyo, Japan). *E. coli *ATCC 25922 and *Pseudomonas aeruginosa *ATCC 27853 were used as quality controls for MIC determination. The Chi-square test was performed on the antimicrobial susceptibility and the phylogenetic subgroup data. The resistance rates in each phylogenetic group were compared with those in the overall strains tested. For each comparison, a *P *value of < 0.05 was considered to denote significant differences.

A double-disk synergy test for detection of ESBLs was performed using clavulanate and cefotaxime, ceftazidime, cefpodoxime, or aztreonam disks (Nissui Pharmaceutical Co., Ltd., Tokyo, Japan) as previously described [10]. Detection of β-lactamase genes was carried out by PCR using primers for *bla_TEM_*, *bla_SHV _*and *bla_PSE-1 _*as described previously [10]. Multiplex PCR was applied to the AmpC genes, groups ACC, FOX, MOX, CIT and EBC, detection as previously described [16]. As for samples positive for CIT group, sequence analysis was performed using the primer pairs for amplification of the full-length gene of *bla_CMY-2 _*[10]. Multiplex PCR was used for *bla*CTX-M detection as previously described [17]. Subtypes of CTX-M β-lactamases were determined using the primer pairs as follows: CTX-MF (5'-GACTATTCATGTTGTTGTTATTTC-3') and CTX-MR (5'-TTACAAACCGTTGGTGACG-3') for CTX-M-group1 [18]; *bla_CTX-M-2_*F (5'-ATGATGACTCAGAGCATTCG-3') and *bla_CTX-M-2_*R (5'-TCAGAAACCGTGGGTTACGA-3') for CTX-M-group2 [10]; CTXM825F (5'-CGCTTTGCCATGTGCAGCACC-3') [19] and CTXM20 (5'-ATAACCGTCGGTGACAATT-3') [17] for CTX-M-group8 and 25/26. Nucleotide sequences were preformed directly on both strands of PCR products by using dye terminator chemistry. The DNA alignments and deduced amino acid sequences were examined using the BLAST program (National Center for Biotechnology Information, USA).

The phylogenetic analysis revealed that 318 isolates of *E. coli *from diseased animals were classified into four phylogenetic groups (Table 1). The phylogenetic group A was commonly predominant in isolates from cattle (36/72, 50%) and pigs (101/157, 64.3%) whereas groups A (44/89, 49.4%) and D (40/89, 44.9%) were predominant in isolates from poultry. The B2 group was rarely found in *E. coli *from healthy cattle, chickens and pigs in Brazil [3] as well as Japan. In Korea, group B2 was also not found in *E. coli *isolates from food-producing animals [4]. These trends were similar to those of the present study although clinical isolates from food-producing animals were used here. On the other hand, in the United States, groups A and D were predominant (38% and 28%, respectively) in *E. coli *from diseased poultry and group B2 was also found in 19% [6]. In European countries, group B2 was often isolated from diseased poultry [7,8]. Thus, the distribution of phylogenetic groups may be determined not only by the animal species but also by their health status or geographical region.

**Table 1:** Distribution of *Escherichia coli *isolate phylogenetic groups from diseased animals in Japan between 2001 and 2006

Phylogenetic group	No. of isolates tested				No. of isolates resistant to cefazolin (%)			
		
	Cattle	Poultry	Swine	Total	Cattle	Poultry	Swine	Total
A	36	44	101	181	3 (8.3)	21 (47.7)	2 (2.0)	26 (14.4)
B1	18	4	9	31	2 (11.1)	1 (25.0)	0 (0)	3 (9.7)
B2	0	1	0	1	0 (NA)	0 (0)	0 (NA*)	0 (0)
D	18	40	47	105	1 (5.6)	7 (17.5)	1 (2.1)	9 (8.6)

Total	72	89	157	318	6 (8.3)	29 (32.6)	3 (1.9)	38 (11.9)

* NA: not applicable

Previous studies showed that the phylogenetic group distribution may be related to antimicrobial resistance prevalence in human isolates of *E. coli *[20]. In the present study, 38 (11.9%) isolates exhibited CEZ resistance (MIC: ≥32 μg/ml) (Table 2). Twenty-six (68.4%) of the CEZ-resistant isolates belonged to group A. However, rates of CEZ resistance among the phylogenetic groups, except for group B2, were in 8.6-14.4%. Thus, no significant difference in the resistance rates between the phylogenetic groups was observed.

**Table 2:** MICs of β-lactam antibiotics for 38 cefazolin-resistant isolates of *E. coli *from diseased animals isolated between 2002 and 2006

					MICs (μg/ml) of:						
					
Animal species	β-lactamase	n	Year isolated	Phylogenetic group	Cefazolin	Cefpodoxime	Cefotaxime	Ceftazidime	Aztreonam	Imipenem	Meropenem
					(32)*	(8)	(64)	(32)	(32)	(16)	(16)
Cattle	CTX-M-2	1	2003 (1)	A (1)	256	>32	>32	4	16	≤1	≤4
	CTX-M-2/TEM-1	1	2003 (1)	B1 (1)	256	>32	>32	8	>16	≤1	≤4
	CMY-2	1	2003 (1)	A (1)	256	8	≤1	≤1	≤4	≤1	≤4
	CMY-2/TEM-1	1	2002 (1)	D (1)	256	>32	8	16	≤4	≤1	≤4
	TEM-1	1	2003 (1)	A (1)	32	8	≤1	2	≤4	≤1	≤4
	ND	1	2002 (1)	B1 (1)	128	16	≤1	8	≤4	≤1	≤4

Poultry	CTX-M-2	1	2006	A (1)	>512	>32	>32	2	≤4	≤1	≤4
	CTX-M-15/TEM-1	1	2006	A (1)	>512	>32	>32	32	8	≤1	≤4
	CTX-M-25	4	2005(3), 2006 (1)	A (2), D (2)	>512	>32	16->32	2-4	≤4-8	≤1	≤4
	SHV-2	3	2004 (3)	A (3)	256-512	32->32	8-32	4-16	≤4-8	≤1	≤4
	CMY-2	10	2004(1), 2006 (10)	A (5), B1 (1), D (5)	512->512	>32	4-32	8-32	≤4-8	≤1	≤4
	TEM-1	7	2004 (2), 2005 (5)	A (7)	64->512	16-32	≤1-2	4-8	≤4	≤1	≤4
	ND	2	2002 (1), 2005 (1)	A (2)	128-256	16-32	≤1-2	4	≤4	≤1	≤4

Swine	CMY-2/TEM-1	1	2004 (1)	A (1)	>512	>32	16	32	16	≤1	≤4
	ND	2	2002 (1), 2003 (1)	A (1), D (1)	128-256	4-8	≤1	≤1	≤4	≤1	≤4

* Resistance breakpoint (μg/ml) defined by CLSI guideline.

Of the 38 resistant isolates, 11 isolates from two cattle and nine poultry produced ESBLs while the remaining 27 isolates produced AmpC β-lactamase. The two bovine isolates expressed *bla_CTX-M-2_*, while, of the nine poultry, four expressed *bla_CTX-M-25_*, three expressed *bla_SHV-2_*, one expressed *bla_CTX-M-15 _*and one expressed *bla_CTX-M-2_*. The *bla_TEM-1 _*gene, being non-ESBL, was detected in 12 isolates. This is the first description of the *bla_SHV-2_*, *bla_CTX-M-15 _*and *bla_CTX-M-25 _*enzymes in *E. coli *from food-producing animals in Japan. In human patients in Japan, *E. coli *strains carrying *bla_CTX-M-2 _*were predominantly prevalent around 1997 [21], and then the dominant β-lactamase type has changed to the CTX-M-9 group, including *bla_CTX-M-14 _*and *bla_CTX-M-16_*, since 2001 [22]. As for food-producing animals, *E. coli *carrying *bla_CTX-M-2 _*have emerged in apparently healthy broilers [10] and cattle [11] in 2000 and pigs in 2003 [12]. In 2002, *bla_CTX-M-18_*, identical to amino acid sequence of *bla_CTX-M-14_*, was identified in cephalosporin-resistant isolates from healthy broilers [10]. On the other hand, of the remaining 27 AmpC β-lactamase producing isolates, 13 isolates were PCR positive for CIT group, being identified *bla_CMY-2_*: 10 from poultry, two from cattle and one from swine (Table 2). There were no positive signals for group ACC, FOX, MOX and EBC. The *bla_CMY-2 _*gene was found to be prevalent in a wide range of food-producing animal species in Japan [10,12], as well as other countries [23].

In the present study, 5 of 8 isolates producing CTX-M ESBL and all three isolates producing SHV-type ESBL belonged to group A. However, group B2 is predominant in SHV-type ESBL producing *E. coli *isolates of human origin whereas group D is predominant in CTX-M enzyme-producing isolates [24]. On the contrary, 7 of 13 isolates producing CMY-2 β-lactamase belonged to group A. Group B1 was dominant in CMY enzyme-producing *E. coli *from retail broiler meats [25]. It is likely that the genotype of *E. coli *carrying β-lactamase genes may be different between humans and food-producing animals.

It is essential to understand the prevalence of resistance to clinically important antimicrobials in bacteria from food-producing animals. The MICs of six β-lactam antibiotics are summarized in Table 2. All CEZ-resistant isolates were susceptible to imipenem and meropenem. All isolates exhibit cefpodoxime resistance (MIC of breakpoint: ≥8 μg/ml) except for one porcine isolate in which β-lactamase expression was not determined. All isolates expressing CTX-M enzymes exhibit cefotaxime resistance (MIC of breakpoint: ≥64 μg/ml) with the exception of one poultry isolate (MIC: 16 μg/ml) harboring *bla_CTX-M-25_*. Resistance to ceftazidime was found in three poultry and one porcine isolate with *bla_CMY-2_*, and one poultry isolate with *bla_CTX-M-15_*. Resistance to aztreonam was found in a single bovine isolate with *bla_CTX-M-2_*. The MICs of antimicrobials tested varied in isolates with each β-lactamase gene.

In conclusion, our study suggests that the distribution of phylogenetic groups in *E. coli *from diseased domestic animals varies regionally in addition to animal species. Moreover, ESBL-producing *E. coli *from the animals were frequently assigned to a specific group, being different from human ESBL isolates. Further study on ESBL-producing *E. coli *from several animal sources using the PCR-based phylogenetic typing and the other genotyping methods may clarify the relationship of the resistant bacteria and/or the resistance determinants prevalent in the food-chain and the environments.

## Abbreviations

CEZ: cefazoline; CLSI: Clinical and Laboratory Standards Institute; ESBL: extended spectrum β-lactamase; ESC: extended-spectrum cephalosporinase; MICs: Minimum inhibitory concentrations.

## Competing interests

The authors declare that they have no competing interests.

## Authors' contributions

TA conceived the study, the study design, interpreted the data and drafted the manuscript. KM carried out large parts of the resistance gene determination. CS helped to carried out resistance gene determination. MH helped to carry out determination of phylogenetic groups. MU helped to carried out resistance gene determination. KB carried out antimicrobial susceptibility testing. MO carried out antimicrobial susceptibility testing. KH carried out antimicrobial susceptibility testing and helped to draft the manuscript. HA helped to carry out the resistance gene determination and draft the manuscript. TS helped to draft the manuscript. All authors read and approved the final manuscript.
